# Pannexin channels mediate the acquisition of myogenic commitment in C_2_C_12_ reserve cells promoted by P2 receptor activation

**DOI:** 10.3389/fcell.2015.00025

**Published:** 2015-05-06

**Authors:** Manuel A. Riquelme, Luis A. Cea, José L. Vega, Carlos Puebla, Aníbal A. Vargas, Kenji F. Shoji, Mario Subiabre, Juan C. Sáez

**Affiliations:** ^1^Departamento de Fisiología, Facultad de Ciencias Biológicas, Pontificia Universidad Católica de ChileSantiago, Chile; ^2^Program of Anatomy and Developmental Biology, Institute of Biomedical Science, Faculty of Medicine, University of ChileSantiago, Chile; ^3^Experimental Physiology Laboratory (EPhyL), Instituto Antofagasta, Universidad de AntofagastaAntofagasta, Chile; ^4^Centro Interdisciplinario de Neurociencias de Valparaíso, Instituto Milenio, Universidad de ValparaísoValparaíso, Chile

**Keywords:** calcium signal, membrane permeability, MyoD, ATP, purinergic receptors, pannexons, myogenesis

## Abstract

The acquisition of myoblast commitment to the myogenic linage requires rises in intracellular free Ca^2+^ concentration ([Ca^2+^]_i_). Putative cell membrane pathways involved in these [Ca^2+^]_i_ increments are P2 receptors (P2Rs) as well as connexin (Cx) and/or pannexin (Panx) hemichannels and channels (Cx HChs and Panx Chs), respectively, which are known to permeate Ca^2+^. Reserve cells (RCs) are uncommitted myoblasts obtained from differentiated C_2_C_12_ cell cultures, which acquire commitment upon replating. Regarding these cells, we found that extracellular ATP increases the [Ca^2+^]_i_ via P2Rs. Moreover, ATP increases the plasma membrane permeability to small molecules and a non-selective membrane current, both of which were inhibited by Cx HCh/Panx1Ch blockers. However, RCs exposed to divalent cation-free saline solution, which is known to activate Cx HChs (but not Panx Chs), did not enhance membrane permeability, thus ruling out the possible involvement of Cx HChs. Moreover, ATP-induced membrane permeability was inhibited with blockers of P2Rs that activate Panx Chs. In addition, exogenous ATP induced the expression of myogenic commitment and increased MyoD levels, which was prevented by the inhibition of P2Rs or knockdown of Panx1 Chs. Similarly, increases in MyoD levels induced by ATP released by RCs were inhibited by Panx Ch/Cx HCh blockers. Myogenic commitment acquisition thus requires a feed-forward mechanism mediated by extracellular ATP, P2Rs, and Panx Chs.

## Introduction

During skeletal muscle ontogeny and regeneration, pluripotential mesodermal or satellite cells acquire myogenic commitment, which involves the expression of myogenic determination factors such as MyoD, Myf-5, and myogenin, transforming these cells into proliferative myoblasts (Charge and Rudnicki, 2004).

The acquisition of myogenic commitment requires increases in intracellular free Ca^2+^ concentration ([Ca^2+^]_i_), which promote the activation of calcineurin (a Ca^2+^-dependent protein phosphatase) that, in turn, induces the expression of the Myf5 transcription factor (Friday and Pavlath, 2001). Increases in [Ca^2+^]_i_ could result from the activation of purinergic P2 receptors (P2Rs) with ATP/ADP, which are divided into two receptor families, namely ionotropic P2X and metabotropic P2Y receptors (P2YRs and P2XRs) (North, 2002; Araya et al., 2004). P2XRs are members of a family of ligand-gated non-selective cationic channels called P2X_1−7_ and are permeable to cations, including Na^+^, K^+^, and Ca^2+^ (North, 2002; Araya et al., 2004). Furthermore, the activation of P2XRs 2, 4, and 7 has been shown to increase cell membrane permeability to small molecules, including Lucifer yellow, ethidium (Etd^+^) and YO-PRO-1 in diverse cell types such as myoblasts and macrophages (North, 2002; Araya et al., 2004; Pelegrin and Surprenant, 2006). However, increase of plasma membrane permeability to dyes induced by P2X_7_R activation is absent in cells lacking pannexin1 (Panx1) expression (Pelegrin and Surprenant, 2006; Locovei et al., 2007), suggesting that channels composed of Panx1 mediate dye uptake induced by P2X_7_R activation. Moreover, activation of Panx1 channels (Panx1 Chs) can also be promoted via P2YRs (Locovei et al., 2006). In addition to Panxs, most cells also express connexins (Cxs), which have been shown to form connexons, also known as Cx hemichannels (Cx HChs). A connexon takes up half of a gap junction channel (GJC) and can be found on the cell surface communicating the intra and extracellular compartments (Sáez et al., 2010) just like Panx Chs do. Panx1 Chs and Cx43 HChs are permeable to Ca^2+^ and small molecules, including signaling molecules such as ATP (Bao et al., 2004; Vanden Abeele et al., 2006; Kang et al., 2008; Schalper et al., 2010). Diverse stimuli can increase the open probability of Cx HChs, including membrane depolarization to positive values, pro-inflammatory conditions, reduced extracellular Ca^2+^ concentration and rises in intracellular [Ca^2+^]_i_, among others (Sáez and Leybaert, 2014).

L6 cells constitute a cell line derived from rat myoblasts. Treating these cells with β-glycyrrhetinic acid, which blocks Cx based GJCs and HChs as well as Panx1 Chs (Schalper et al., 2008a), has been shown to inhibit the expression of myogenin and MRF4, two transcription factors that promote myogenesis and inhibits the cellular fusion process that leads to myotubes formation (Proulx et al., 1997). However, treatment with octanol, which is a blocker of Cx GJCs and HChs, but not Panx1 HChs (Bruzzone et al., 2003; Pelegrin and Surprenant, 2006), does not affect myogenesis as evaluated through the expression of the pro-myogenic transcription factor Myf5 (Proulx et al., 1997). These findings suggest that Cx GJCs and HChs are not involved in the acquisition of myogenic commitment. Accordingly, it was recently reported that Panx1 channels favor the differentiation of skeletal muscles, since inhibition of Panx1 Chs drastically reduces differentiation, whereas overexpression of Panx1 enhances muscle differentiation (Langlois et al., 2014). Muscle differentiation is a high-order process involving several steps that begin with the acquisition of myogenic commitment and the identification of the specific step affected by Panx1 Chs remains to be elucidated.

The roles of Panx1 Chs and Cx HChs in numerous biological responses in the absence of GJCs (e.g., lack of GJC expression or low density cultures that prevent GJC formation) can be distinguished by using pharmacological approaches. Panx Chs are relatively insensitive to several Cx HCh blockers, including octanol, heptanol, flufenamic acid, and La^3+^ (Bruzzone et al., 2003; Pelegrin and Surprenant, 2006). Moreover, Panx1 Ch activity is insensitive to reductions in extracellular [Ca^2+^] (Bruzzone et al., 2003; Locovei et al., 2006; Ma et al., 2009), while Cx HChs are activated in cells bathed with saline solutions containing low extracellular concentrations of divalent cations (Ca^2+^ and Mg^2+^) (Schalper et al., 2008a).

In C_2_C_12_ cell cultures, the fusion process reaches its maximum at around day 6 post-induction of differentiation. At that time, these cultures contain fused myoblasts called myotubes and undifferentiated mononuclear cells, called reserve cells (RCs), with undetectable levels of MyoD and Myf5 (Yoshida et al., 1998). In differentiated cultures containing myotubes and RCs, the extracellular activity of α-sarcoglycan, which is an ectoATPase, is also high and is likely to explain the low extracellular ATP levels present in the extracellular solution (Sandona et al., 2004). C_2_C_12_ RCs isolated by controlled trypsinization and re-plating with serum-rich medium have been shown to acquire myogenic commitment (Yoshida et al., 1998).

The present work was mainly undertaken to demonstrate the possible role of extracellular ATP, P2 receptors, Cx HChs and Panx1 Chs during the acquisition of myogenic commitment in C_2_C_12_ RCs. This molecular trilogy was found to play a critical role in skeletal muscle commitment acquisition by C_2_C_12_ RCs.

## Materials and methods

### Reagents

An affinity purified polyclonal anti-Panx1 serum developed in chicken was purchased from Diateva (Roma, Italy). Polyclonal antibody directed against the whole MyoD molecule was acquired from Santa Cruz Biotechnology (Santa Cruz, CA, USA), and monoclonal antibodies anti-LAP2 was purchased from Transduction Laboratories (Louisville, KY, USA). The adenosine 5′-triphosphate bioluminescence assay, ethidium bromide (Etd^+^), suramin, oleamide, oxidized ATP (oATP), carbenoxolone (CBX), 18 β-glycyrrhetinic acid (β-GA), FITC-conjugated goat anti-rabbit IgGs, and TRITC conjugated goat anti-mouse IgGs were obtained from Sigma (St. Louis, MO, USA). Enhanced chemiluminescence (ECL) reagents were from Pierce Biotechnology (Piscataway, NJ, USA). MRS2179 was obtained from TOCRIS (Park Elisville, MO, USA) and pyridoxalphosphate-6-azophenyl-2′,5′-disulphonate (iso-PPADS) was purchased from Cookson (Southampton, UK). Panx1 siRNA and its control (FlexiTube GeneSolution, cat n° ID: 2120593) were obtained from Qiagen (Germantown, MD, USA). Lipofectamine LTX and PLUS Reagent (cat n° 15338100) and Opti-MEM (cat n° 31985-070) were from Life Technologies (Carlsbad, CA, USA). pEGFP-N1 vector was obtained from Clontech Laboratories (Mountain View, CA, USA).

### Cell lines, culture of C_2_C_12_ cells, and isolation of RCs

The C_2_C_12_ cell line derived from mouse skeletal muscle (ATCC, Manassas, VA, USA) was grown as described by Araya et al. (2004). Briefly, cells (22 × 10^4^) were seeded on tissue culture dishes of 100-mm diameter (CORNING, Garden Glove, CA, USA) containing growth medium (GM: DMEM/F12 supplemented with 10% FBS, and 100 U/ml penicillin, 100 μg/ml streptomycin). After 3 days in GM, cell differentiation was triggered by replacing GM with differentiation medium (DM: DMEM/F12 medium supplemented with 5% horse serum, 100 U/ml of penicillin, and 100 μg/ml streptomycin). Cell cultures were fed every 48 h with DM. At day 10 of culture in DM, RCs were isolated as described by Yoshida et al. (1998). At that time period cell cultures were subjected to controlled trypsinization and released cells were seeded, 1 h after which they were washed three times and fed with GM in which RCs become myoblasts indicating acquisition of myogenic commitment (Yoshida et al., 1998).

### Transfection

C_2_C_12_ cells were transfected with Panx1 siRNA at final concentration of 100 nM using Lipofectamine LTX and PLUS Reagents as described for the 35 mm dish format according to the manufacturer's instructions. The transfection was performed 24 h before isolation of RCs. After 28–30 h of transfection cells were used for MyoD detection or Etd^+^ uptake experiments. Also the cells were transfected with pEGFP-N1 vector to control the transfection efficiency.

### Electrophysiology

Electrophysiological measurements were carried out in subconfluent cell cultures plated on glass coverslips (#1) containing numerous single cells. Two hours after plating, coverlips containing cells were transferred to an experimental chamber mounted on the stage of an inverted microscope (Olympus IX-51, Olympus Optical Co, NY). For whole-cell experiments the bath solution contained (in mM) 140 NaCl, 5.4 KCl, 1 MgCl_2_, 1.8 CaCl_2_, 2 BaCl_2_, 10 Hepes, pH 7.4 and the pipette solution contained (in mM) 130 CsCl, 10 AspNa, 0.26 CaCl_2_, 1 MgCl_2_, 2 EGTA, 7 TEA-Cl, 5 Hepes, pH 7.2. Patch pipettes were made from borosilicate glass capillaries using a flaming/brown micropipette puller (P-87, Sutter Instruments CO, Union City, CA, USA). The tip resistance was 5–10 MΩ when filled with pipette solution. Whole-cell currents were recorded by using either voltage ramps or voltage steps increasing in 20 mV from −80 to +80 mV, as described previously (Schalper et al., 2008b). Currents were filtered at 1 kHz and sampled at 5 kHz. Then, records were filtered with a digital low-pass filter of 0.5 kHz. Data acquisition and analysis were performed with pClamp 9 (Axon Instruments, Novato, CA, USA).

### Dye uptake and [Ca^2+^]_i_ measurements

For dye uptake measurements, RCs were plated onto glass coverslips and after 2 h they were washed twice with Krebs–Ringer buffered saline solution (in mM: 145 NaCl, 5 KCl, 1 CaCl_2_, 1 MgCl_2_, 5.6 glucose, 10 HEPES-Na, pH 7.4) containing 5 μM Etd^+^, and fluorescence was recorded at regions of interest in different cells with a water immersion Olympus 51W1I upright microscope. Images were captured with a Q Imaging model Retiga 13001 fast-cooled monochromatic digital camera (12-bit) (Qimaging, Burnaby, BC, Canada) every 30 s (exposure time = 30 ms, gain = 0.5) and image processing was performed off-line with ImageJ software (NIH, Bethesda, USA).

The Ca^2+^/Mg2^+^-free saline contained (in mM) 145 NaCl, 5 KCl, 0.5 EGTA, 5.6 glucose, 10 HEPES-Na, pH 7.4. Cells seeded on glass coverslips were placed in a 1 ml chamber located on the stage of an inverted microscope equipped with epifluorescence illumination (Olympus T041, New Hyde Park, NY, USA), where recordings were performed. After excitation with a 488-nm wavelength with a Xenon arc lamp and filter system, the fluorescence sequences of 80 images were collected every 4.5 s at 200-ms exposure. Data were acquired with a CCD cooled camera (MCD600, Spectra Source Instruments, West lake Village, CA, USA) connected to a microscope side port; the full or partial image acquisition was computer-controlled through macros that operate the software provided by the manufacturer. Image processing was done off-line with the public domain ImageJ software. The collected data were illustrated as folds of basal fluorescence vs. the difference of initial fluorescence and fluorescence at the time of interest (Δ*F*/*F*_0_) - (Δ*F*/*F*_0_)*b*, corrected with respect to basal fluorescence in order to reduce the photo bleaching artifact of Fluo-3.

### Western blot and indirect immunofluorescence analysis

Cells were washed twice with ice-cold PBS (pH 7.4) and then harvested by scraping with a rubber policeman in 1 ml lysis buffer (PBS containing protease inhibitors: 2 mM phenylmethylsulfonyl fluoride, 200 μg soybean trypsin protease inhibitor, 1 mg/ml benzamidine, 1 mg/ml ε-aminocaproic acid, and 500 μg/ml leupeptin, and phosphatase inhibitors: 20 mM Na_4_P_2_0_7_ and 100 mM NaF) and then sonicated. Western blot analyses were performed as described previously (Schalper et al., 2008b). Blots were incubated overnight with polyclonal rabbit immunopurified anti-MyoD antibodies diluted with 5% non-fat milk in PBS. Then, they were rinsed with PBS and incubated for 1 h at room temperature with horse radish peroxidase-conjugated goat anti-rabbit IgG antibodies at appropriated dilution in PBS with 5% non-fat milk in PBS. After repeated rinses, immunoreactive proteins were detected using ECL reagents (Pierce biotechnology, Rockford, IL) according to the manufacturer's instructions.

Cells grown on glass coverslips were washed three times with PBS, pH 7.4, fixed with 4% formaldehyde for 5 min at room temperature and then incubated in blocking solution (PBS–1% BSA, pH 7.4) for 30 min at room temperature. Samples were incubated overnight at 4°C with appropriately diluted rabbit anti-MyoD antibody. Then, samples were processed as previously described (Araya et al., 2004). Immunoreactive sites were detected with FITC-conjugated goat anti-rabbit IgG secondary antibodies. Cells were rinsed and mounted with fluoromount G (Electron Microscopy Sciences, Hatfield, PA, USA) on glass slides and observed under a Nikon Labophot-2 microscope equipped with epifluorescent illumination and photographed. Immunolocalization of MyoD was carried out in coverslips and mounted in Vectashield (Vector Laboratories) for confocal microscopy and representative images were acquired (Carl Zeiss Axiovert 135, LSM Microsystems). Only secondary antibodies were added for negative controls.

### Data analysis and statistics

For each data group, results are expressed as mean ± SEM, and *n* refers to the number of independent experiments. For statistical analysis, each treatment was compared to its respective control, and significance was determined by using a One-Way ANOVA followed by a Tukey *post-hoc* test. Differences were considered significant at *p* < 0.05. Statistics were performed with Microsoft Excel (2007) and Graph Pad Prism 4 (2003).

## Results

### Extracellular ATP activates Ca^2+^ signal in uncommitted RCs via purinergic receptors and panx channels

In other cell types, extracellular ATP increases the [Ca^2+^]_i_ through activation of P2X or P2Y receptors (Illes and Alexandre Ribeiro, 2004). Moreover, activation of P2Rs has been demonstrated to be required for skeletal muscle terminal differentiation (Araya et al., 2004). Here, we evaluated whether P2Rs are present and participate in commitment acquisition, which is an earlier stage of skeletal muscle ontogeny. To address this issue, uncommitted RCs (Yoshida et al., 1998) were obtained from differentiated cultures of C_2_C_12_ cells. They were loaded with the free Ca^2+^ indicator Fluo-3 and stimulated with a bath application of ATP, while the (Δ*F*/*F*_0_) - (Δ*F*/*F*_0_)*b* (hereinafter called Ca^2+^ signal) was monitored.

**Figure 1:**
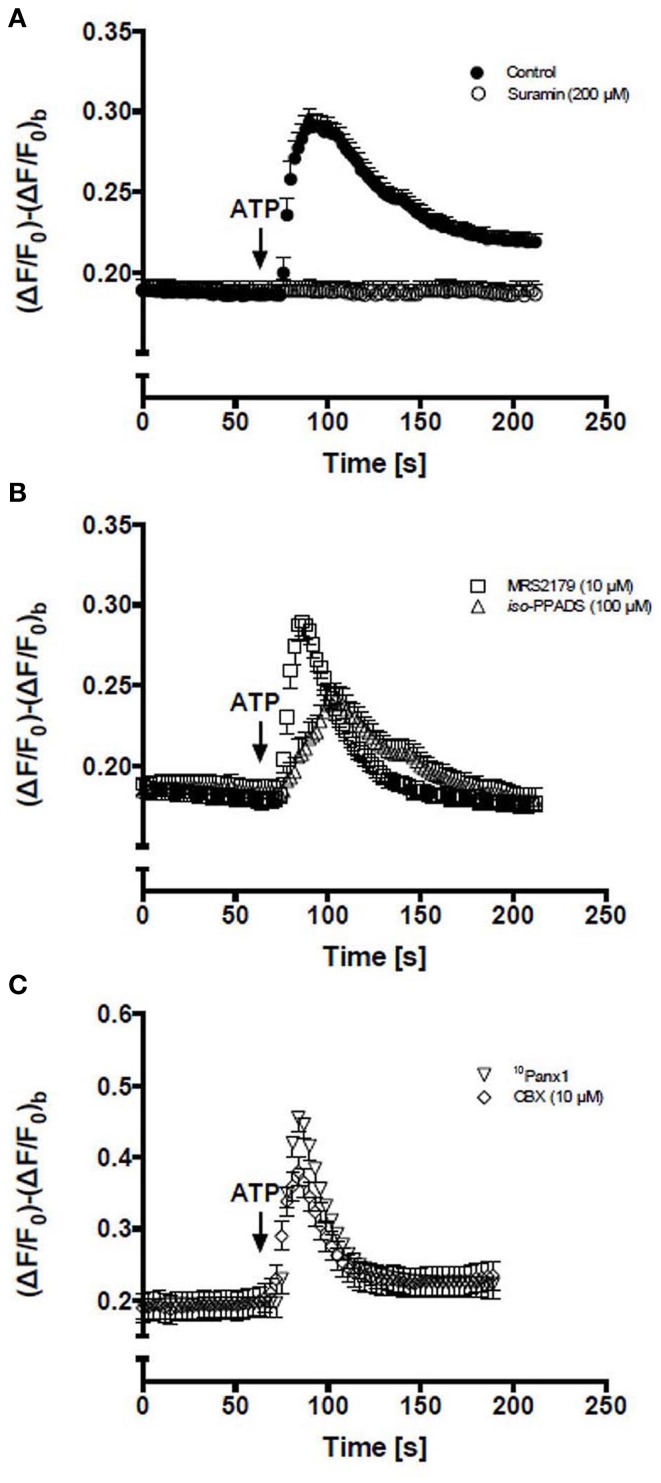
**Activation of P2Rs with extracellular ATP elevates intracellular Ca^2+^ levels in C_2_C_12_ RCs**. Time course of relative fluorescence changes induced by bath application of 150 μM ATP (arrow) in C_2_C_12_ RCs loaded with Fluo-3. **(A)** Cells were stimulated with 150 μM ATP alone or preincubated (5 min) with 200 μM suramin (a P2Y and P2X receptor antagonist) and then treated with ATP. **(B)** RCs were preincubated (5 min) with 10 μM MRS2179 or 100 μM iso-PPADS followed by stimulation with 150 μM ATP. **(C)** RCs preincubated (5 min) with ^10^Panx1 peptide or 10 μM carbenoxolone (CBX) and then stimulated with ATP. Each point corresponds to the mean ± SEM, *n* = 3 experiments. At least 10 cells were recorded per experiment.

After the application of 150 μM ATP, the Ca^2+^ signal in all cells remained unchanged for a brief period of time (<10 s) and then showed a rapid increase followed by a plateau phase well-above the basal value (Figure 1A). To determine the contribution of P2Rs, RCs were pretreated for 5 min with 200 μM suramin, which is a concentration that blocks P2XRs and P2YRs, and then treated with 150 μM ATP, resulting in absence of Ca^2+^ signal response (Figure 1A). Then, the possible contribution of ionotropic P2XRs and metabotropic P2Y_1_R in the Ca^2+^ signal triggered by ATP was evaluated. Cells were pre-treated for 5 min with 100 μM iso-PPDAS, a general P2XR blocker (Araya et al., 2004) or 10 μM MRS2179, a specific P2Y_1_R blocker (Baurand and Gachet, 2003). The inhibition of P2XRs drastically reduced the amplitude and raise phase of the transient Ca^2+^ signal peak; the response showed only ~34% amplitude and ~24% area under the curve as compared to control (Figure 1B), while these two parameters were not significantly affected by P2Y_1_R inhibition (Figure 1B). Notably, the plateau phase that followed the transient peak of Ca^2+^ signal promoted by extracellular 150 μM ATP was completely abrogated by the inhibition of P2XRs or P2Y_1_Rs (Figure 1B), suggesting that iso-PPDAS-sensitive P2XRs and MRS2179-sensitive P2Y_1_Rs are partially responsible for activating the mechanism that drives the plateau phase of the Ca^2+^ signal. In cells pretreated with 150 μM oATP, another P2XR blocker, the Ca^2+^ signal elicited by 150 μM ATP was comparable to that of cells pretreated with iso-PPDAS (not shown).

In order to study the possible involvement of Panx1 Chs in Ca^2+^ signaling promoted by extracellular 150 μM ATP, RCs were first treated for 5 min with 200 μM ^10^Panx1 peptide or 10 μM carbenoxolone, which are two Panx1 Ch blockers (Bruzzone et al., 2005; Pelegrin and Surprenant, 2006). Under these conditions, the Ca^2+^ signal elicited by RCs was fast and transient but was not followed by a persistent plateau phase (Figure 1C), suggesting a critical involvement of Panx1 Chs in the establishment of this feature of the ATP-promoted Ca^2+^ signal in RCs.

### RCs present membrane panx channels activated by extracellular ATP

Extracellular ATP also increases membrane permeability to small molecules mainly through activation of Panx1 Chs (Locovei et al., 2006; Pelegrin and Surprenant, 2006; Nishida et al., 2008). In the present study, ATP increased [Ca^2+^]_i_ in C_2_C_12_ RCs (Figure 1), but it remained unknown whether RCs exhibit active Cx HChs or Panx Chs at the cell surface.

**Figure 2:**
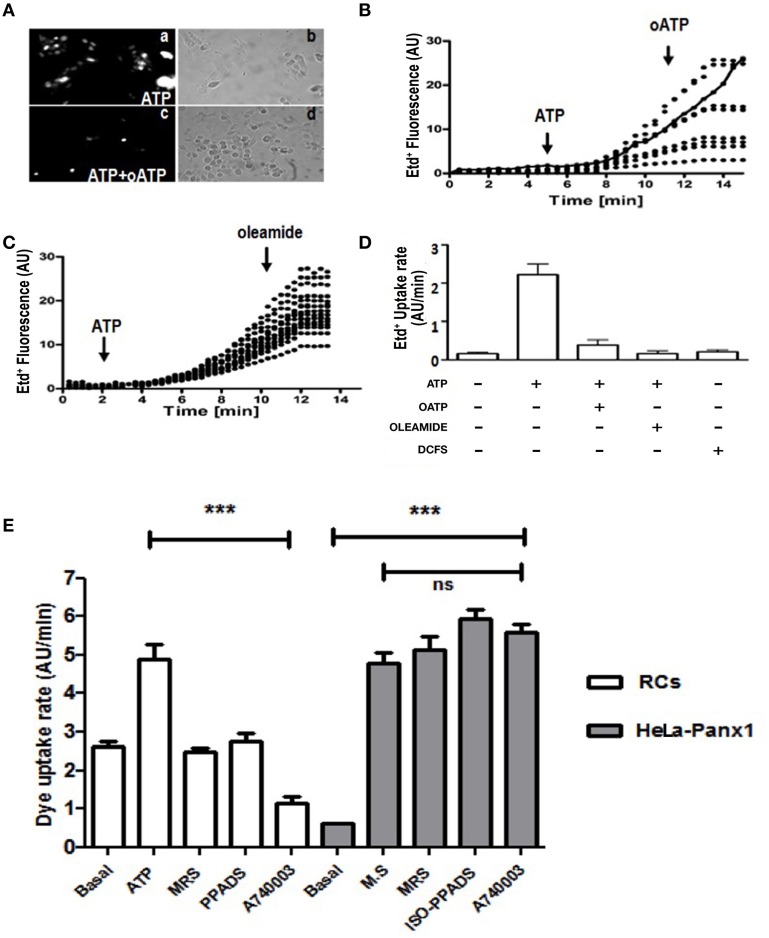
**C_2_C_12_ RCs express functional P2X receptors and pannexin channels**. C_2_C_12_ RCs seeded onto glass cover slips were maintained for 1 h in growth medium so they could attach. Then, cells were washed three times and after 0.5 h they were exposed to Krebs–Ringer saline solution containing 5 μM Ethidium (Etd^+^). Thereafter, cells were treated with 150 μM ATP and Etd^+^ uptake was evaluated. **(Aa)** Microphotograph taken 15 min after 150 μM ATP application. **(Ac)** co-application of 150 μM oATP blocked dye uptake induced by 150 μM ATP. (**Ab**, **Ad**) Phase contrast views of fields shown in **Aa** and **Ac**, respectively. **(B)** Time-lapse measurements of Etd^+^ uptake in several cells (8 in **B** and 14 in **C**) under control conditions (5 or 2 min, respectively) and after the application of 150 μM ATP **(B, C)**, indicated with the arrow. After 10 or 8 min of recording under control conditions 150 μM oATP **(B)** or 100 μM oleamide **(C)** was applied (arrow). In **(B)**, the continuous line represents the mean ± SEM of three experiments in cells treated only with ATP. **(D)** Bar graph showing the Etd^+^ uptake rate of cells treated as in (**B**) and **(C)**. Each number corresponds to the average ± SEM (*n* = 3 experiments); 8–15 cells were recorded per experiment. In addition, cells were exposed to saline solution without Ca^2+^ and Mg^2+^ (DCFS), known to induce Cx HC opening. **(E)** Etd^+^ uptake rate in RCs treated with ATP or in HeLa-Panx1 cells treated with mechanical stress (M.S.) to induce opening of Panx1 channels. In both cell types the effect of blockade of P2Y_1_R (30 μM MRS2179), P2XRs (50 μM iso-PPADS) or P2X_7_R (10 μM A740003) on the Etd^+^ uptake rate was evaluated. ^***^p < 0.001.

To demonstrate the presence of Panx Chs activated by extracellular ATP via P2Rs in C_2_C_12_RCs, we evaluated changes in membrane permeability to Etd^+^, which has been used as a permeability probe in time lapse measurements (Schalper et al., 2008a,b). Treatment with 150 μM ATP for 15 min induced a heterogeneous Etd^+^ uptake response of RCs (Figure 2Aa). To determine if the ATP-induced response was mediated by P2XRs, RCs were simultaneously treated for 15 min with 150 μM oATP and 150 μM ATP. Under this condition, no Etd^+^ uptake was detected in ~90% of the cells (Figure 2Ac). Then, the ATP-induced Etd^+^ uptake was quantified over time. Etd^+^ uptake was very low during the first 2–4 min of recording under control conditions (Figures 2B,C). However, at about 4 min after treatment with 150 μM ATP, a rapid increase in Etd^+^ uptake occurred (Figures 2B,C) and either the acute application of 150 μM oATP (Figures 2B,D) or 100 μM oleamide (Figures 2C,D) drastically reduced Etd^+^ uptake, thus reaching values close to those measured under control conditions. Treatment of RCs with 150 μM ATP induced Etd^+^ uptake as described above and the acute application of selective a P2Y_1_R (MRS2179) (Baurand and Gachet, 2003), P2XRs (iso-PPADS) or P2X_7_R (A740003) (Honore et al., 2006) blocker drastically reduced Etd^+^ uptake (Figure 2E), suggesting that simultaneous ATP-induced activation of P2Y and P2X receptors would be required for opening of Panx1 Chs in RCs. Moreover, we tested whether P2Y_1_R and P2X_7_R blockers affect the activity of open Panx1 Chs. To this end, HeLa cells transfected with Panx1 were mechanically stressed with eight drops of saline solution falling from about 10 cm high to induce Panx1 Ch opening. During recordings of Etd^+^ uptake, cells were treated with MRS2179, iso-PPADS or A740003, which did not affect the Etd^+^ uptake rate (Figure 2E), indicating that these compounds do not block Panx1 Chs.

Divalent cation free solution (DCFS), known to increase the open probability of Cx HChs, did not promote Etd^+^ uptake in RCs (Figure 2D), suggesting that Cx HChs are not involved in ATP induced Etd^+^ uptake.

To further demonstrate the presence of Panx Chs in cell membranes of RCs, we characterized the membrane current responses induced by transmembrane voltages under resting conditions and after applying extracellular ATP in the absence and presence of Cx HCh/Panx1 Ch blockers, which only blocked Panx Chs in this preparation because we did not detect Cx HChs (see above).

**Figure 3:**
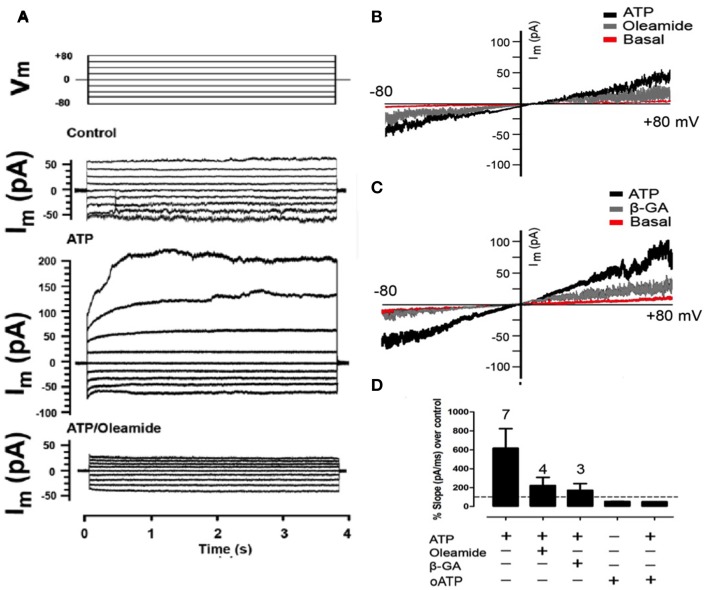
**Extracellular ATP enhances the membrane current mediated by Panx1 channels**. Representative I/V curve of total membrane current recorded in freshly seeded RCs evaluated under voltage clamp by using the whole cell patch clamp configuration. Rectangular voltage steps or voltage ramp between −80 and +80 mV were applied during ~4 or 20 s, respectively. **(A)** Top traces correspond to the protocol of voltage commands (Vm) applied and the other traces correspond to a representative set of currents recorded under control conditions, after treatment with 300 μM ATP or simultaneous treatment with 100 μM oleamide and 300 μM ATP. **(B,C)** The membrane current promoted by voltage ramp under resting conditions (Basal), followed by the application of 150 μM ATP and then, the application of **(A)** 100 μM oleamide or **(B)** 50 μM β-GA. **(D)** Bar graph showing the percentage with respect to control conditions of the slope of membrane currents, in the presence (+) or absence (–) of ATP, 100 μM oleamide, 50 μM β-GA as described above or 150 μM oATP in the absence or presence of 150 μM ATP. The digit above each bar indicates the number of experiments.

Two hours after plating, total RC membrane current was evaluated by means of whole-cell patch clamp and applying voltage steps (~4 s and 20 mV changes) or ramps between −80 and +80 mV of 5 s duration. Under this condition, the membrane currents generated at all voltages were very small (Figures 3A–C) and increased linearly passing through zero at 0 mV (Figures 3B,C). In less than 10 s treatment with 150 μM ATP, the currents generated with different voltage commands were much more robust and the I/V curves showed an increase in total current as compared to control conditions (Figures 3A–C). In addition, after treatment with 100 μM oleamide (Figures 3A,B) or 50 μM β-GA (Figure 3C) total current drastically decreased to values close to or even below those recorded under basal conditions.

After 150 μM ATP treatment, the slope of the membrane current trace was 617 ± 207% above that of control conditions (Figure 3D). In the same RC treatment the application of 100 μM oleamide reduced ATP-induced current to 224 ± 86% above control conditions (Figure 3D). Similarly and in separate experiments, the slope of the ATP-induced membrane current was reduced to 172 ± 71% above control conditions in RCs treated with 50 μM β-GA (Figure 3D). Surprisingly, the application of 150 μM oATP, which is a P2XR blocker (Araya et al., 2004), reduced the current slope to 50 ± 1% below that of RCs under control conditions (not treated with ATP) (Figure 3D), suggesting the involvement of functional P2XRs under basal activations. Moreover, the application of 150 μM oATP immediately reduced the slope of the ATP-induced current to 53 ± 2% below control values (Figure 3D).

### Acquisition of myogenic commitment requires activation of P2X receptors and functional panx1 channels

**Figure 4:**
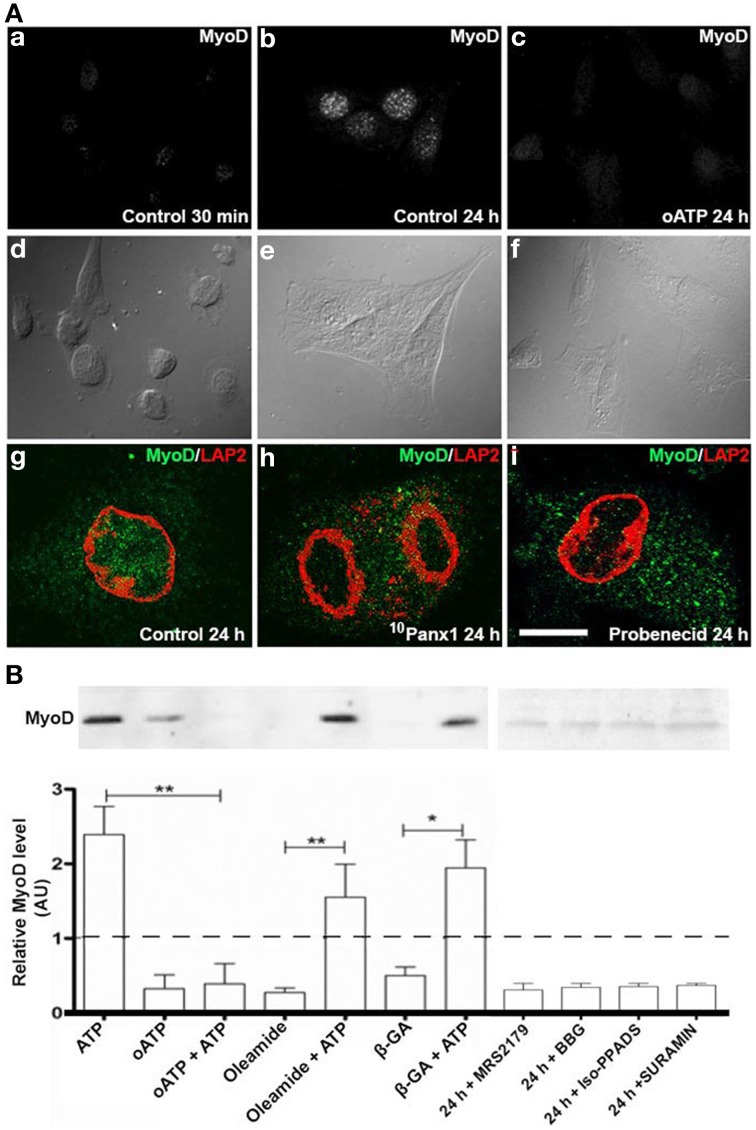
**C_2_C_12_ RCs require functional P2X receptors and Panx1 channels for myogenic commitment acquisition. (A)** Immunofluorescence detection of MyoD in C_2_C_12_ RCs cultured for 0.5 h **(Aa)** and 24 h in **(Ab)** the absence or **(Ac)** presence of 150 mM oATP. **Ad–Af** are contrast phase views of fluorescent fields shown in **Aa–Ac**, respectively. Bar = 20 nm. **Ag–Ai** show the presence of MyoD (green) in nuclei, marked by LAP2 (red), a nuclear membrane marker, at 24 h of RC cultures, in normal **(Ag)**, siPanx1 **(Ah)**, and probenecid (**Ai**, 1 mM) treated cells. **(B)** Two consolidates of at least three Western blot analyses of C_2_C_12_ RCs under control conditions (24 h) or after treatment for 24 h with 300 μM ATP, 150 μM oATP, 30 μM MRS2179, 200 μM brilliant blue G (BBG), 50 μM iso-PPADS, 200 μM suramin or HC blockers added simultaneously: 50 μM b-GA, 100 μM oleamide in the absence or presence of 300 μM ATP. All values were normalized to the control value of each experiment. ^*^p < 0.05 and ^**^p < 0.01.

MyoD levels increase in C_2_C_12_ RCs cultured in GM, hence revealing the acquisition of myogenic commitment (Yoshida et al., 1998). To determine the role of P2Rs in this process, immunofluorescence and Western blot analyses of MyoD in RCs treated with different P2R and Panx1 Ch inhibitors were performed (Figure 4). Since primary cultures of RCs were contaminated with differentiated myotubes that express MyoD, the possible role of P2 receptors on MyoD expression by RCs was first evaluated by immunofluorescence detection in isolated mononuclear cells at different time periods after plating. MyoD was not detected in any of the mononuclear cells at 0.5 h after plating, indicating that they were uncommitted RCs (Figure 4Aa). However, after 24 h all RCs showed MyoD reactivity (Figures 4Ab,Ag) and the increased expression of MyoD was completely prevented in all RCs bathed in GM containing 150 μM oATP (Figure 4Ac), 200 μM ^10^Panx1 (Figure 4Ah) or 1 mM probenecid (Figure 4Ai).

As seen through Western blot analyses, MyoD levels in cells treated with 300 μM ATP were ~2.5 fold higher than in control cells (basal MyoD levels found in total cell homogenates could be explained by the contaminating myotubes mentioned above and were considered as basal levels in normalization). Additionally, MyoD levels in RC cultures treated with 300 μM oATP were even lower than in control cultures (Figure 4B). Since the P2XR blocker drastically reduced ATP-induced dye uptake (Figure 2B), it was possible to infer that oATP blocked ATP release, and thus, the extracellular ATP concentration necessary to effectively activate P2Rs was not attained. To test this possibility, RCs were treated simultaneously with 300 μM oATP and exogenous 300 μM ATP. Under these conditions, the effect of oATP predominated over the effect of endogenous ATP (Figure 4B), indicating the absolute requirement of functional P2XRs in order to transduce the action of extracellular ATP in controlling MyoD levels. Since ATP can be released to the extracellular milieu through Panx1 Chs, the effects of oleamide and β-GA (two Panx1 Ch blockers) were tested in relation to increases in MyoD levels induced by exogenous ATP. MyoD levels in RCs treated with 100 μM oleamide or 50 μM β-GA were lower than in control cells (Figure 4B), suggesting that Panx Chs play a relevant role in this process. However, the presence of Panx Ch blockers did not significantly affect the increase in MyoD levels induced by exogenous ATP (Figure 4B), suggesting that the release of endogenous ATP via Panx Chs was overcame by the added ATP. Moreover, inhibition of P2Y_1_R (MRS2179), Panx1 Chs (oleamide), P2Xs and Panx1 Chs (BBG), P2XRs (iso-PPADS or oATP) and P2YRs/P2XRs (suramin) reduced MyoD levels to values below those found in RCs cultured in GM (Figure 4B).

**Figure 5:**
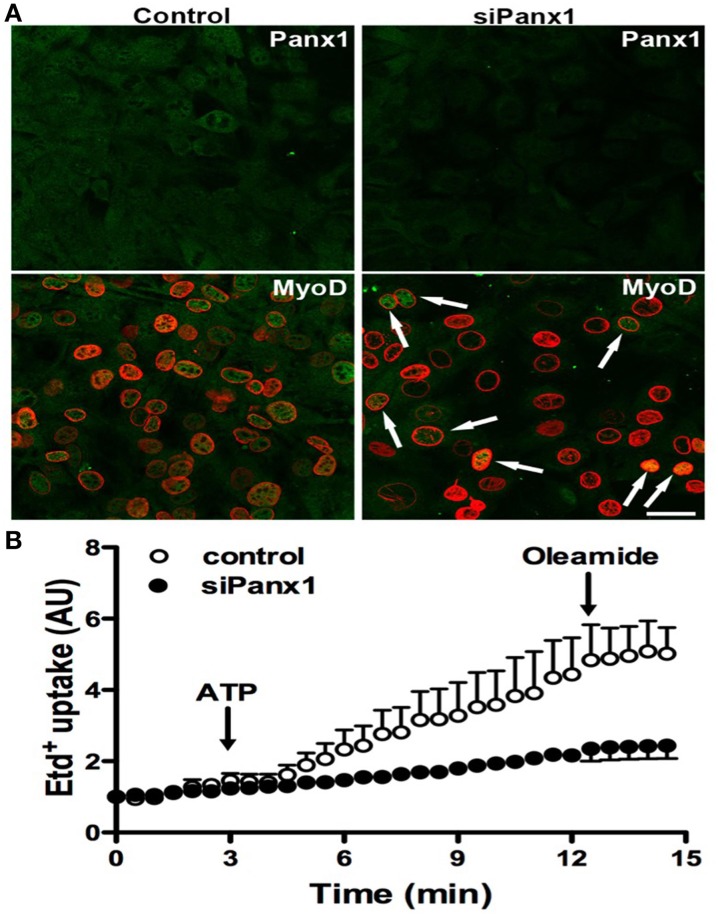
**Panx1 is required for ATP-induced myogenic commitment of RCs**. Reserve cells (RCs) were isolated from 7 days in differentiated C_2_C_12_ cultures and placed on cover slips. For siPanx1 experiments, C_2_C_12_ differentiated cultures were transfected with siPanx1 in lipofectamine solution at 24 h before isolation of RCs. After 24 h of isolation of RCs (48 h of siPanx1 transfection) Panx1 reactivity (Green, top panels) was reduced (Top right) as compared to control condition (top left). The MyoD nuclear distribution was analyzed (**A**, green) by co-immunofluorescence with LAP2 (red signal), which is a nuclear membrane marker. In parallel experiments, hemichannel activity was monitored in ethidium (Etd^+^) uptake assays (**B**), where Etd^+^ uptake was induced with ATP (150 μM) and inhibited with oleamide (100 μM).

To further demonstrate the importance of Panx1 Chs in myogenesis, we studied whether Panx1 turndown affects MyoD activation by using immunofluorescence and confocal microscopy. After 48 h of induced the acquisition of myogenic commitment with GM, all control cells presented MyoD reactivity in the nucleus underlined with Lap2, which is a nuclear membrane marker (Figure 5A). However, ~75% of the cells transfected with siRNA for Panx1 did not present MyoD reactivity in the nucleus (Figure 5A). The efficiency of transfection was tested with the same transfection protocol and a vector carrying the cDNA for a fluorescent protein (pEGFP) corresponded to ~75% (not shown). In addition, ATP-induced Etd^+^ uptake of cells transfected with siRNA for Panx1 was drastically reduced as compared to untransfected cells (Figure 5B).

### Acquisition of myogenic commitment occurs in the absence of connexin gap junctions

**Figure 6:**
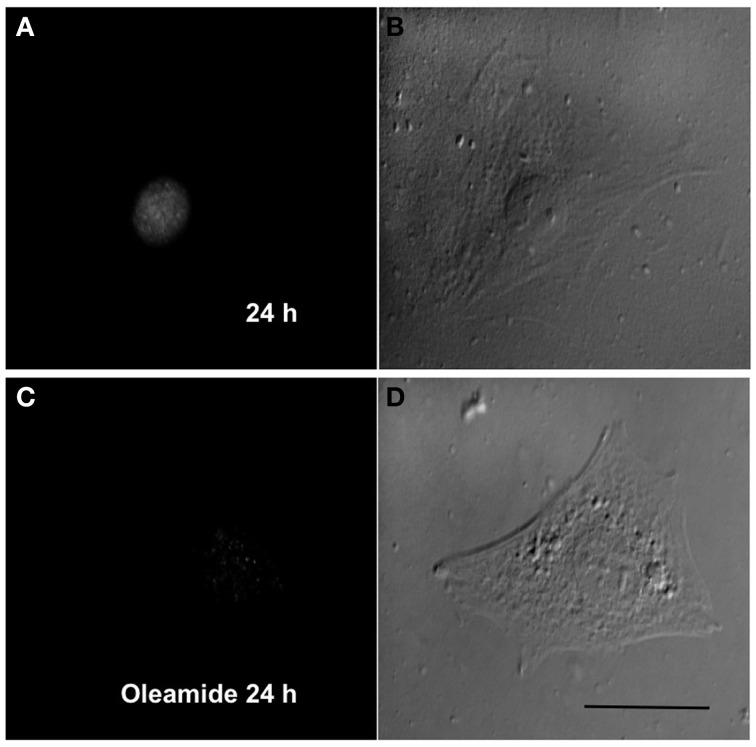
**Panx1 channels, but not gap junction channels, are needed for myogenic commitment acquisition of RCs**. Sparse reserve cells (RCs), to avoid gap junction formation, were cultured under control conditions **(A)** or in the presence of 100 μM oleamide **(C)**, which is a Panx1 channel blocker. After 24 h of culture, cells were fixed and processed for MyoD detection by immunofluorescence. Panels **(B)** and **(D)** are phase contrast views of the fluorescent fields shown in **(A)** and **(C)**, respectively.

Since gap junction channels have been proposed to play a relevant role in the late stages of myogenic differentiation (Araya et al., 2004), we decided to study whether gap junctional communication is required for an early steps such as toward the myogenic commitment response. This possibility was tested in RCs cultured in low density to avoid the formation of cell–cell contacts where gap junction channels can be formed. Under these conditions, mononucleated cells without physical contact with neighboring cells cultured for 24 in GM medium presented MyoD reactivity in the nucleus (Figures 6A,B). In contrast, cells cultured in GM containing 100 μM oleamide (to block Panx1 Chs) did not present MyoD reactivity (Figures 6C,D).

## Discussion

In this report, we observed that extracellular ATP increases the Ca^2+^ signal via P2Rs as well as membrane current and permeability to Etd^+^ mediated by Panx1 Chs and expression levels of MyoD in C_2_C_12_ RCs. Moreover, we showed that RCs do not express functional Cx HChs on their surface, but express Panx1 Chs that possibly serve to release ATP to the extracellular milieu. Therefore, we propose that the acquisition of myogenic commitment in RCs requires a feed forward mechanism that includes Panx Ch-dependent ATP release and activation of P2Rs.

We found that only a few seconds (<10 s) after ATP application RCs showed a rise in Ca^2+^ signal and an increase in total membrane current sensitive to Cx HCh/Panx Ch blockers. The rapid and transient rise in Ca^2+^ signal was mediated by P2Y and P2X receptors, since it was only partly prevented by the inhibition of P2XRs with iso-PPDAS and oATP (two P2XR blockers) or MRS2179 (P2Y_1_R blocker), and was completely prevented by suramin, which is a non-selective blocker of both P2YRs and P2XRs (Illes and Alexandre Ribeiro, 2004). On the other hand, the late increase in Ca^2+^ levels induced by ATP was not prevented by either of the two P2 receptor blockers used, but further studies would be required to elucidate the mechanism involved. Moreover, freshly seeded RCs treated with ATP showed a rapid (<10 s) increase in total membrane current mediated by Panx Chs, since it was reduced by oleamide and β-GA and Cx HCh activity was not detected in DCFS. This is in agreement with previous results indicating that octanol, a Cx based channel blocker, does not affect myogenesis (Proulx et al., 1997). However, Cx43-based channels have been observed in transgenic mice with an inducible CRE-lox (p) system to abrogate the expression of Cx43^*flox/flox*^ (Araya et al., 2003, 2005). In this system, the regeneration of skeletal muscle after injury is delayed with respect to wild type animals by about 14 days, suggesting that myoblasts require cellular coordination via Cx43 membrane channels for a normal timing of regeneration.

A fraction of the ATP-induced membrane current appears to be mediated by P2XRs, since the current slope was smaller in RCs treated with oATP alone than in RCs under resting conditions. Moreover, an important part of the ATP-induced current increase (>63%) was mediated by HCs, since both oleamide and β-GA reduced the total membrane current slope to a value about twice of that recorded in control cells and three times lower than that measured after treatment with ATP alone. Similar activation of Panx Chs via P2XRs has been demonstrated in other cell types (Locovei et al., 2006, 2007; Pelegrin and Surprenant, 2006). P2XRs belong to an ionotropic membrane receptor family (North, 2002). If P2X_7_Rs were the only P2XRs expressed by RCs, the heterogeneity of the Etd^+^ uptake response elicited by ATP might be explained by the heterogeneous P2X_7_R abundance. Moreover, the P2YR type expressed by RCs is likely to be P2Y_1_R, since MRS2179, which is a selective P2Y_1_R blocker (Baurand and Gachet, 2003), reduced or inhibited several RC responses described herein.

With regard to the possible molecular composition of HCs involved in both ATP-induced increases in membrane current and membrane permeability to Etd^+^, RCs did not express functional Cx HChs as indicated by the lack of dye uptake after exposure to DCFS, which is known to activate Cx HChs and not Panx Chs (Schalper et al., 2008a; Ma et al., 2009). An alternative explanation for the inhibitory effect of Cx HCh blockers on the effects of ATP described herein would be the existence of Panx Chs. Whether RCs express other members of the Panx family remains unknown, and therefore, demonstration of monomeric or heteromeric Panx Chs in RCs would require further investigation.

Previous studies have demonstrated the autocrine/paracrine role of ATP during skeletal muscle differentiation (Ryten et al., 2002; Araya et al., 2004) and ATP release through Panx1 Chs was shown to elicit Ca^2+^ signals involved in gene expression in rat myotubes (Buvinic et al., 2009). The present study found that RCs release ATP to the extracellular milieu, which most likely occurred via Panx1 Chs because channel blockers or the Panx1 knockdown drastically reduced the acquisition of myogenic commitment.

During skeletal muscle embryogenesis, somites express transcription factors that control different processes, including migration and myogenic transcription factors that regulate terminal differentiation such as MyoD, Myf-5, and myogenin (Charge and Rudnicki, 2004). Moreover, the role of P2X receptors play a role in proliferation and/or differentiation of skeletal muscles (Burnstock et al., 2013). In agreement with this interpretation, blockade of P2XRs with oATP, even when co-added with ATP, prevented the increase of MyoD levels, suggesting that it is required for the acquisition of myogenic commitment. In line with this notion, oleamide and β-GA prevented the rise of MyoD levels. However, this inhibition was totally reversed with the addition of ATP, showing that these Cx HCh/Panx Ch blockers are not toxic and do not block purinergic pathways.

The results described in the present work might contribute to explaining findings in other preparations. For instance, treatment of primary myoblasts with α-GA inhibits the increase of MyoD and increases levels of adiposity markers PPARγ and C/EBPα, both of which are transcription factors required for adipose differentiation (Yamanouchi et al., 2007), suggesting that Cx and/or Panx HCs play relevant roles during those events. In support of this possibility, Panx3 has been proposed to form GJCs between osteoblasts and to contribute to the differentiation of C_2_C_12_ cells into osteoblasts (Ishikawa et al., 2011).

Despite the absence of Panx1 in knockout mice, these animals have no apparent phenotype due to deficient skeletal muscle myogenesis. A possible explanation might be that in absence of Panx1 (a Ca^2+^ channel) myocytes express other Ca^2+^-permeable channel as a compensatory mechanism. In this sense, it has been established that a compensatory increase of P2X_7_R (i.e., a Ca^2+^ channel) expression occurs in lymphocytes from Panx1 knockout mice (Shoji et al., 2014), which also might occur in skeletal muscles. However, there are still some physiological issues regarding skeletal muscles from Panx1 knockout animals. For instance, these muscles do not produce potentiation of muscular contraction (Riquelme et al., 2013). Furthermore, additional problems in these mice have been published, such as uncompleted abolishment (70%) of Panx1 mRNA in some tissues like trigeminal ganglia, bladder and spleen (Hanstein et al., 2013). Additionally, it has been established in different systems that Panx1 Ch activation requires functional P2X or P2YR upstream Panx1 Ch activation, but in this case it was necessary to simultaneously stimulate both P2 receptor types, which could be explained by two manners of Panx1 Ch activation. The first one is mediated by activation of Ca^2+^ inflow from the extracellular space through P2XRs and the other is mediated by G-protein signaling and Ca^2+^ from intracellular stores induced by P2YR activation. This possibility implies that activation of both P2 receptors leads to an optimal [Ca^2+^]_i_ for Panx1 HC activation, but neither P2XR nor P2Y_1_R alone would be sufficient.

Finally, we propose that purinergic P2Rs and Panx1 Chs are part of a positive feedback system present in C_2_C_12_ RCs. Activation of P2XRs by extracellular ATP, released through Panx Chs activated via P2Rs. Moreover, activated P2XRs and Panx Chs are permeable to Ca^2+^ (Vanden Abeele et al., 2006) and, thus, they might contribute to the rises in Ca^2+^ signals observed in ATP-treated RCs. As a result of the latter, Panx1 Chs could be activated via a cytoplasmic factor (i.e., PKC and/or calmodulin/Ca^2+^-dependent kinase) (Barbe et al., 2006), allowing for more ATP release. The positive loop may be inhibited in differentiated cultures of C_2_C_12_, which could provide a possible explication for the reduction of MyoD levels in mononucleated cells, since the extracellular medium is known to contain high levels of phosphatase activity (Sandona et al., 2004). The latter is directly related to the expression of α-sarcoglycan, which is a proteoglycan with ATP binding domains and phosphatase activity (Sandona et al., 2004). In this way, both the ATP tone and MyoD levels could be diminished. In support of this putative mechanism, replated RCs with low levels of myotube contamination, and consequently, low levels of phosphatases, would allow for ATP accumulation in the extracellular medium, which would induce the acquisition of myogenic commitment.

### Conflict of interest statement

The authors declare that the research was conducted in the absence of any commercial or financial relationships that could be construed as a potential conflict of interest.
